# Identifying the ideal weekly training load for in-game performance in an elite Brazilian soccer team

**DOI:** 10.3389/fphys.2024.1341791

**Published:** 2024-03-05

**Authors:** Luís Branquinho, Elias de França, José E. Teixeira, Adriano Titton, Luís Fernando Leite de Barros, Pedro Campos, Daniel A. Marinho, Pedro Forte, Erico Chagas Caperuto, Ronaldo Vagner Thomatieli dos Santos, Ricardo Ferraz

**Affiliations:** ^1^ Agrarian School of Elvas, Polytechnic Institute of Portalegre, Portalegre, Portugal; ^2^ CI-ISCE–ISCE Douro, Penafiel, Portugal; ^3^ Research Center in Sports Sciences, Health Sciences and Human Development, Covilhã, Portugal; ^4^ Department of Biosciences, Federal University of São Paulo, São Paulo, Brazil; ^5^ Sport Sciences and Physical Education Department, Polytechnic Institute of Bragança, Bragança, Portugal; ^6^ Sport Department, Polytechnic Institute of Guarda, Guarda, Portugal; ^7^ São Paulo Futebol Clube, São Paulo, Brazil; ^8^ University of Beira Interior, Covilhã, Portugal; ^9^ Sport Department, Higher Institute of Educational Sciences of the Douro, Penafiel, Portugal; ^10^ LiveWell—Research Centre for Active Living and Wellbeing, Polytechnic Institute of Bragança, Bragança, Portugal; ^11^ Human Movement Laboratory, São Judas University, São Paulo, Brazil

**Keywords:** soccer, fixture congestion, match demands, training, load

## Abstract

**Introduction:** The purpose of this study was to investigate the ideal training load to be applied during periods of fixture congestion to ensure an adequate dose-response effect for performance maintenance.

**Methods:** Match performance data and corresponding pre-match training load sessions (both N = 498 match performance cases and training-block session cases) were collected (with the catapult system, VECTOR7) from 36 male professional soccer players (23.5 ± 5.2 years; 178 ± 4 cm; 75.5 ± 6.0 kg) belonging to the Brazilian First Division team during the 2022 season. The following data were collected in match and training sessions: jump, acceleration, deceleration, and change of direction (COD); running distance producing metabolic power at different intensities (>20, >20–35, >35–45, >45–55, and >55 W kg^−1^), total distance (m), relative distance (m/min), running distance at different speeds (>20, >25, and >30 km/h), number of sprints (running >25 km/h), and maximum speed (km/h). Mixed linear model (MLM), decision tree regression (DTR), and cluster K means model (SPSS v.26) approach were performed to identify the most critical variables (and their respective load) in the training sessions that could explain the athlete’s match performance.

**Results:** MLM and DTR regression show that training load significantly affects game performance in a specific way. According to the present data, an interference phenomenon can occur when a high load of two different skills (running in a straight line vs COD, deceleration, and jumping) is applied in the same training block of the week. The cluster approach, followed by a chi-squared test, identified significant associations between training load and athlete match performance in a dose-dependent manner.

**Discussion:** The high load values described here have a beneficial effect on match performance, despite the interference between stimuli discussed above. We present a positive training load from a congested season from the Brazilian First Division team. The study suggests that an interference effect occurs when high physical training loads are applied to different specific physical skills throughout the season.

## 1 Introduction

In recent years, there has been a growing interest in the detailed analysis of training and match demands in professional soccer (Sarmento et al., 2018; Clemente et al., 2019). This is partly due to the development of GPS and tracking systems that provide detailed performance analysis (i.e., training load) (Kaloop et al., 2017; Reinhardt et al., 2019).

Data collected during training and match sessions are essential to characterize match demands, define optimal training loads in preparation for competitions (Gómez-Carmona et al., 2018; Gonçalves et al., 2022), detect fatigue patterns (Filetti et al., 2019), prevent injuries, reduce the risk of overtraining (Rodrigues et al., 2023), and provide a broader understanding of each player’s profile (Carling et al., 2008; Reche-Soto et al., 2019).

Modern elite soccer involves a large number of competitions and matches (typically up to 50 games) during the season between national and international competitions (Thorpe and Sunderland, 2012). Thus, it is not uncommon for a team to play two matches in a single week (Julian et al., 2021) with little recovery time in between, which represents a congested schedule. Indeed, the ability to recover between matches and intense training has previously been identified as a determinant of success (Rey et al., 2018; Dolci et al., 2020). Therefore, an approach that identifies the optimal training load and potential overload between games could be crucial for coaches.

Indeed, during periods of fixture congestion, the maintenance or improvement of performance is determined not only by adequate conditioning, but also by the ability of body systems to recover and regenerate after multiple stress stimuli (Marqués-Jiménez et al., 2017; Kalkhoven et al., 2021). Previous evidence has reported that reducing recovery time between games can lead to residual fatigue (Lago-Peñas et al., 2011), increase player stress, increase the risk of injury, and impair performance (Dellal et al., 2015; Mohr et al., 2016). Thus, in championships that face long and congested schedules with little recovery time (e.g., Brazil’s Serie A) (Vieira et al., 2018), the coach’s strategies in squad rotation and the application of appropriate training loads in a training context are particularly important.

Training load can be identified with a dose-response relationship between training stimuli and changes in physical fitness indicators (Branquinho et al., 2021a; Branquinho et al., 2021b), which have been widely used to identify peaks in training load. Monitoring the training load can be essential to optimize performance, reduce the risk of injury, and give the coach a general idea of how weekly stimuli affect performance in a game (Borin et al., 2007; Guerrero-Calderón et al., 2021; Guerrero-Calderón et al., 2022).

The training load is usually planned to ensure that the player is available for the next match (Garcia et al., 2022). However, in leagues (e.g., Brazil’s Serie A) with a tight schedule and long travel times between games, training time is reduced. For these reasons, it is essential that the stimuli applied (i.e., training load) do not exceed recommended levels, yet little is known about the ideal training load for teams facing these challenges over the course of a season. In fact, the management and control of training load (i.e., internal load and external load) throughout the weekly periodization, if done correctly, can be critical in ensuring that players arrive at the next game in the best possible condition (Teixeira et al., 2021; Teixeira et al., 2022). New information on this topic would be of great use to coaches and sports scientists in optimizing player performance during the season.

Thus, the main objective of this study was to investigate the ideal training load to be applied during periods of fixture congestion to ensure the appropriate dose-response effect for performance maintenance. Our central hypothesis is that the weekly training load in a congested schedule is strongly associated with match performance. Also, there is an association between match performance and multiple contextual factors (such as home-away match condition, player position, amounts of training sessions in the weekly macrocycles, and the days between games). Finally, the type of stimulus applied in the macrocycles (such as jumping stimulus, change of direction, the number of explosive actions, and running in a straight line) might exert a positive or negative influence depending on the match performance variables assessed.

## 2 Material and methods

### 2.1 Participants and sample

**FIGURE 1:**
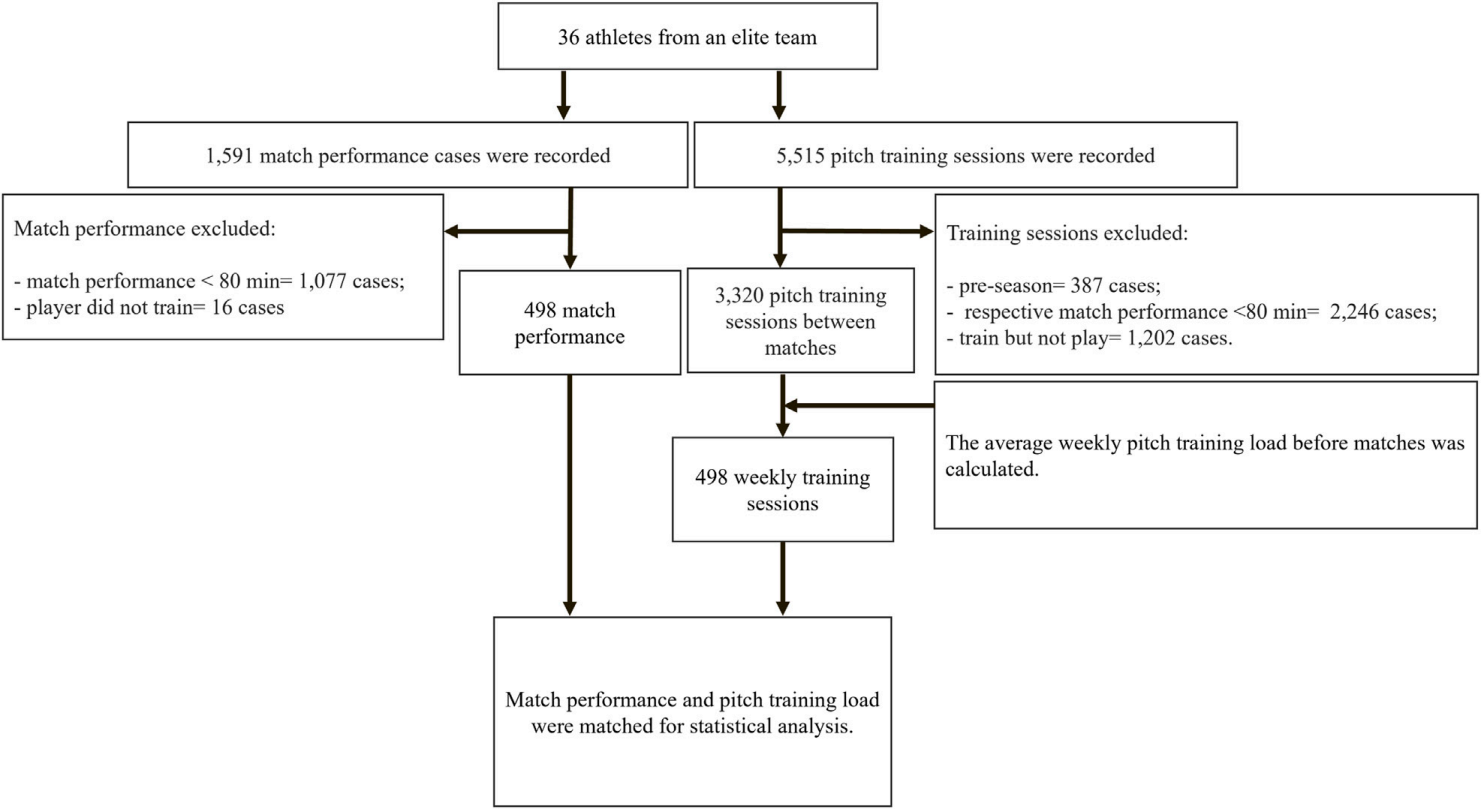
Flowchart with inclusion and exclusion criteria for match performance and pitch training data.

Match performance data (N = 1,596 cases) and pitch match training load sessions (N = 5,515 cases) were collected from 36 male professional soccer players (age 23.5 ± 5.2 yr; 178 ±4 cm; 75.5 ± 6.0 kg) belonging to the Brazilian First Division team during the 2022 season. Only data corresponding to 77 official matches from 2022 season were analyzed. The 2022 season (with first official match) started on January 27th and ended on November 11th, without breaks during this entire period (that is, with one or two matches every week). Only match performances lasting ≥80 min were included in the analysis in accordance with previous recommendations (Guerrero-Calderón et al., 2021; Guerrero-Calderón et al., 2022). In this sense, the sample was limited to 498 match performance cases and their corresponding previous training load cases. Thus, 1,077 match performances cases and their corresponding previous training load (N = 2,446 cases) with match duration ≤79 min were excluded, see Figure 1. Players were divided into four positions: striker (112 pitch training sessions and match performance cases), fullback (117 cases), winger (88 cases), and midfielder (181 cases), see detailed description in Table 1. Goalkeepers were excluded from this analysis due to the different nature of their movement pattern.

**TABLE 1:** Match performance and pitch training load description according to player position.

	Match performance				*p*-value	Training load				*p*-value
Player position	Striker	Fullback	Winger	Midfield		Striker	Fullback	Winger	Midfield	
**Total distance (m)**	9890.7 (9484.5/10296.8)	10157.6 (9799.3/10515.8) &	9686.2 (9268.5/10103.9)	10432.2 (10135.3/10729.1) #	**0.025**	3199.5 (2896.1/3502.8)	2817.7 (2556.7/3078.7) &	3237.7 (2929.3/3546.1)	3302.3 (3083.0/3521.5) #	**0.032**
**Relative distance (m/min)**	98.4 (95.3/101.5) #	92.6 (89.9/95.3) *, &	94.0 (90.8/97.2) &	100.3 (98.1/102.6) #, @	**0.004**	64.2 (60.2/68.1)	63.5 (60.0/67.0)	64.9 (60.8/68.9)	65.1 (62.3/68.0)	0.876
**Distance >20 km/h (m)**	568.0 (488.3/647.7) #	422.1 (356.3/487.8) *, &	513.7 (434.2/593.3)	566.8 (509.4/624.4) #	**0.033**	174.1 (136.9/211.2)	104.6 (74.9/134.2)	151.1 (114.8/187.3)	143.4 (116.7/170.1)	**0.031**
**Distance >25 km/h (m)**	177.2 (135.5/219.0) #	102.3 (69.2/135.4) *	124.3 (83.9/164.8)	144.0 (114.0/174.1)	**0.033**	48.1 (34.1/62.1) #	21.7 (10.9/32.5) *	35.7 (22.5/49.0)	28.8 (18.7/38.8)	**0.017**
**Distance >30 km/h (m)**	20.8 (11.2/30.5)	11.5 (4.1/18.9)	7.9 (0.0/17.00)	12.6 (5.7/19.5)	0.275	5.7 (2.9/8.6) #	0.7 (0/2.8) *	3.2 (0.7/5.8)	1.3 (0/3.3)	**0.016**
**Sprints (N)**	12.4 (9.8/15.1)#	7.7 (5.6/9.8)*	8.9 (6.3/11.4)	9.9 (8.0/11.8)	**0.045**	3.7 (2.7/4.8) #	1.7 (0.9/2.5) *	2.9 (1.9/3.9)	2.2 (1.4/2.9)	**0.010**
**Max. speed (km/h)**	30.2 (29.5/30.8)	30.1 (29.5/30.6)	29.9 (29.3/30.6)	29.9 (29.4/30.4)	8.54	27 (25.9/28.0)	25.3 (24.4/26.2) @	27.3 (26.2/28.4) #	26.5 (25.7/27.3)	**0.018**
**Accelerations (N)**	18.5 (15.0/22.1)	17.2 (14.2/20.1)	14.0 (10.1/17.6)	13.5 (11.0/16.1)	**0.037**	5 (3.9/6)	4.6 (3.7/5.4)	5.7 (4.7/6.8)	5 (4.3/5.8)	0.332
**Decelerations (N)**	15.7 (12.6/19.2)	20.2 (17.4/23.1)	16.7 (13.3/20.1)	17.4 (14.9/19.9)	0.119	7.4 (5.9/8.8)	6.7 (5.5/8.0)	7.6 (6.2/9.1)	6.7 (5.7/7.8)	0.662
**Jumps > 40 cm (N)**	3.0 (1.8/4.3) #	5.4 (4.4/6.5) *, @, &	2.6 (1.4/3.9)	2.1 (1.2/3.0)	**<0.001**	0.8 (0.4/1.2)	0.5 (0.2/0.8)	0.6 (0.2/1)	0.5 (0.3/0.8)	0.503
**COD to left (N)**	20.2 (16.2/24.2)	21.1 (17.9/24.4)	15.9 (11.9/19.8)	18.1 (15.2/21.0)	0.087	7.4 (5.9/9)	7 (5.6/8.3)	6.2 (4.6/7.8)	7.7 (6.6/8.9)	0.424
**COD to right (N)**	20.6 (17.8/23.3)	21.2 (18.9/23.6)	18.6 (15.8/21.4)	20.3 (18.3/22.3)	0.500	8.6 (6.4/10.8)	7.5 (5.8/9.3)	8.9 (6.8/11)	9.1 (7.5/10.6)	0.387
**Explosive effort**	67.9 (59.8/76.0) #	84.3 (77.7/91.0) #, &	65.5 (57.6/73.6) #	70.71 (64.9/76.5) #	**<0.001**	29 (23.7/34.4)	26 (21.6/30.4)	29.3 (24.0/34.7)	29.2 (25.3/33.1)	0.543
**RHIE blocks effort (N)**	24.2 (21.7/26.6)	23.2 (21.1/25.3)	20.6 (18.2/23.1)	22.5 (20.8/24.3)	0.191	9 (7/11)	6 (4.4/7.6)	8.3 (6.4/10.2)	7.9 (6.5/9.4)	**0.029**
**Average RHIE effort (N)**	4.5 (4.4/4.7)	4.5 (4.3/4.6)	4.4 (4.3/4.5)	4.4 (4.3/4.5)	0.375	4 (3.4/4.7)	4.4 (3.8/5)	4.1 (3.4/4.8)	4.1 (3.6/4.6)	0.844
**RHIE block recovery time (RHIE/min)**	3.4 (3.0/3.9) @	4.2 (3.8/4.5)	4.4 (3.9/4.8) *	3.9 (3.6/4.2)	**0.023**	3.5 (2.9/4)	3.6 (3.1/4.1)	3.7 (3.2/4.3)	3.4 (3.1/3.8	8.35
**Distance > 20w (m)**	2008.4 (1818.4/2198.3)	1975.2 (1815.1/2135.2) &	2031.2 (1839.4/2223.0)	2309.8 (2172.4/2447.2) #	**0.004**	684.2 (587.9/780.4)	544 (464.0/624.0) &	675.2 (578.7/771.7)	729.2 (659.6/798.7) #	**0.004**
**Distance > 55w (m)**	250.6 (217.9/283.2) #	192.6 (165.7/219.5) *	194.1 (161.5/226.7)	195.7 (172.1/219.2)	**0.034**	101.9 (80.7/123.2) #	54.5 (38.0/70.9) *, @	85.8 (65.7/105.9) #	76.1 (60.9/91.3)	**0.001**
**Player load**	1063.5 (982.3/1144.8)	999.1 (933.3/1064.8)	1018.9 (938.8/1099.0)	1035.4 (976.9/1094.0)	0.628	350.3 (313.4/387.2)	310.2 (278.6/341.9) @	373.5 (336.0/411.1) #	347.5 (320.8/374.2)	**0.052**

Data are mean and IC, 95%. The mixed linear model was used to compare match performance and training load across player positions. For this, player ID, was used as a random effect, and player position as a fixed effect. *, *p* < 0,05 when compared to striker; #, *p* < 0,05 when compared to fullback; @, *p* < 0,05 when compared to winger; &, *p* < 0,05 when compared to midfield. Bold values indicate *p* < 0.05.

### 2.2 Data collection

The catapult system (VECTOR7) with global and local positioning system devices (GPS, GLONASS and SBAS 18 Hz; LPS, Catapult ClearSky 10 Hz) combined with inertial sensors such as accelerometer (3D ± 16G; sampled at 1kHz, provided at 100 Hz), gyroscope (3D 2000°/second @ 100 Hz), and magnetometer (3D ±4,900 µT @100 Hz) were used to collect data for all games. All three inertial sensors collected data on acceleration, force, rotation, and body orientation.

### 2.3 Match performance and session training variables

The Inertial Movement Analysis (IMA) method was used to access explosive efforts such as jumps (>40 cm), acceleration (−45 to 0, 0–45°), deceleration (135–180, -180 to −135°), change of direction (COD) to the left (−135 to −45°) or to the right (45–135°). In addition, the total explosive effort (the sum of the jump, acceleration, deceleration, and COD) was recorded as the IMA explosive effort. IMA was used to derive RHIE (Repeat High-Intensity Efforts: the player performed three explosive efforts in ≤60 s) and RHIE block recovery time (the amount of time to recover and perform another RHIE). The running distance producing metabolic power (W kg^−1^) was also collected at different intensities (>20, >20–35, >35–45, >45–55, and >55 W kg^−1^). The player load was collected as the sum of the accelerations of the tri-axial accelerometer. GPS methods were used to collect total distance (m), relative distance (m/min), running distance >20 km/h (m), >25 km/h (m), and >30 km/h. In addition, the number of sprints (running >25 km/h) and the maximum speed (km/h) achieved during the match or training session were recorded.

### 2.4 Training data collection

Training and conditioning sessions between games were collected. Pre-season training was excluded from the analysis. 5,512 training sessions were collected. Only the training load corresponding to players who played in competitive matches for more than >80 min was included. The average number of training sessions per week before the game (reported here as weekly training sessions) was calculated. Players who did not complete a conditioning session but played a match were excluded from the analysis (see detailed description in Figure 1). Strength training and recovery sessions were not included in the analysis.

### 2.5 Contextual factors

Number of training sessions between matches (players are prevented from playing to safeguard themselves for more important matches and only accumulate training sessions to recover); the amount of weekly training (the number of training sessions before a match) and days between matches.

### 2.6 Statistical analysis

Data are presented as mean and standard deviation (SD) (or, when indicated, confidence interval (CI) 95% or minimum a maximum). To determine the influence of training load on match performance throughout the season, the mixed linear model regression fit was used for 19 dependent variables (see Table 2). Two contextual factors were used as fixed effects: player position and match location. Three contextual factors were used as covariables: training sessions between matches, weekly training sessions, and days between matches, and 17 variables were collected during the training session (see Table 2). Collinearity for the inclusion of independent variables was set at a tolerance of >0.1. Because data from the same player were used multiple times, players were used as random effects (intercept model). A decision tree regression (DTR) model was performed to identify the most important variables (and their respective load) in the training sessions that could explain the athlete’s match performance. The DTR model was generated [using the Chi-square Automatic Interaction Detector (CHAID) method]. The nodes (leaves) of the DTR were cleared to compare at least 100 samples in each node. To examine the association between player performance and training session load, the match performance and training session load data were standardized to z-scores and then clustered using a k-means approach. To validate the clusters (i.e., to verify if clusters are different between then), we use the ANOVA-way (using clusters as factors and the z-score of training load variables or match performance as dependent variables). The Chi-squared was used to test the association between the clusters created by k-means and with contextual variables (player position and home-away match condition). All analyses were performed using the statistical package IBM SPSS Statistics v.26.0.

**TABLE 2:** Mixed linear model regression of the variables related to the physical performance of 77 matches (2022 season) of an elite team in Brazil.

Contextual factors and variables from training sessions	Total distance (m)	Relative distance (m/min)	Distance >20 km/h (m)	Distance >25 km/h (m)	Distance >30 km/h (m)	Sprints (N)	Max. Speed (km/h)	Accelerations (N)	Decelerations (N)
Intercept	**12248,3 (10.436.0/13.841.5)**	**107.7 (95.8/118.3)**	**634.3 (411.4/857.2)**	**164.1 (68.2/259.9)**	19.1 (−.8 39.0)	**13.5 (7.4/19.5)**	**30.6 (28.5/32.9)**	**21.7 (11.8/31.7)**	**20.1 (10.4/29.8)**
Match avenue	**−330.8 (-596.4/-87.4)**	**−2.9 (-4.6/-1.3)**	**−59.0 (-91.8/-26.2)**	**−17.9 (-32.1/-3.7)**	−2.5 (−5.3 .4)	−1.0 (−1.9/-0.1)	−0.25 (−0.6/0.1)	0.4 (−1.0/1.8)	−0.4 (−1.8/1.0)
Player position	69.8 (−95.8/227.9)	1.0 (−0.2/2.2)	16.5 (−8.2/41.2)	−.4 (−10.7/9.9)	−1.6 (−4.3 1.1)	−0.2 (−0.8/0.4)	0.03 (−0.2/0.2)	**−1.9 (-3.2/-0.7)**	−0.6 (−1.9/0.7)
Days between games	−57.9 (−136.4/22.5)	**−0.6 (-1.1/-0.1**	**−11.0 (-21.3/-0.6)**	**−4.8 (-9.3–0.4)**	−0.7 (−1.7 .2)	**−0.4 (-0.6/-0.1)**	−0.03 (−0.1/0.1)	0.06 (−0.4/0.5)	−0.2 (−0.6/0.3)
Weekly training session	−99.6 (−264.8/67.5)	−0.6 (−1.7/0.5)	−9.2 (−30.8 12.3)	−3.0 (−12.4/6.3)	−0.7 (−2.6 1.2)	−0.5 (−1.1/0.1)	−0.11 (−0.3/0.1)	0.06 (−0.9/1.0)	−0.2 (−1.1/0.8)
Training sessions between matches	**120.04 (60.6/179.1)**	**0.5 (0.1/0.9)**	**14.5 (6.8/22.1)**	**5.5 (2.2/8.9)**	**1.1(.5 1.8)**	**0.4** (0.2/0.6)	0.07 (−0.01/0.15)	0.01 (−0.3/0.3)	0.1 (−0.2/0.5)
Total distance (m)	−0.1 (−0.6/0.3)	0.0 (0.00/0.00)	−0.04 (−0.1/0.0)	0.0 (0.0/0.0)	0.00 (0.0/0.0)	0.00 (0.0/0.0)	0.00 (−0.01/0.02)	−0.00 (0.0/0.0)	0.00 (0.00/0.00)
Relative distance (m/min)	**−14.8 (-27.7/-1.2)**	−0.1 (−0.15/0.00)	−1.0 (0–2.6/0.9)	−.1 (−0.8/0.7)	0.1 (−0.1/0.2	−0.01 (−0.1/0.0)	0.00 (−0.01/0.00)	0.02 (−0.1/0.1)	0.03 (−0.04/0.10)
Distance >20w (m)	**3.2 (1.2/5.2)**	**0.02 (0.00/0.03)**	**0.35 (0.1/0.6)**	.1 (0.0/0.2)	0.00 (0.0/0.0	0.00 (0.0/0.0)	**−0.00 (0.00/0.02)**	0.00 (0.0/0.0)	0.01 (0.00/0.02)
Distance >55w (m)	**−11.7 (-20.4/-3.2)**	**−0.07 (-.012/-0.01)**	0.58 (−0.6 1/0.7)	.4 (−0.1/0.9)	**0.2 (0.1/0.3**	0.03 (0.0/0.1)	**0.01 (0.00/0.02)**	−0.02 (−01/0.0)	−0.02 (−0.06/0.03)
Distance >20 km/h (m)	**−4.7 (-9.3/-0.5)**	−0.01 (−0.04/0.02)	−0.5 (−1.1/0.0)	−.2 (−0.4/0.1)	−0.01 (−0.1/0.0	−0.01 (0.0/0.0)	0.00 (0.00/0.01)	0.01 (0.0/0.0)	0.00 (−0.03/0.02)
Distance >25 km/h (m)	**24.9 (6.1/43.3)**	0.1 (−0.02 .22)	**4.2 (1.7/6.5)**	.9 (−0.1/1.9)	0.1 (−0.1/0.3	0.06 (0.0/0.1)	0.01 (−0.02/0.03)	0.07 (0.0/0.2)	0.03 (−0.07/0.13)
Distance >30 km/h (m)	6.1 (−31.2/43.2)	0.2 (−0.07/0.42)	0.4 (−4.5/5.2)	.1 (−2.0/2.1)	0.03 (−0.4/0 .5	0.02 (−0.1/0.2)	0.00 (−0.1/0.0)	−0.2 (−0.4/0.0)	−0.1 (−0.3/0.2)
Sprints (N)	−41.3 (−284.5/203.7)	−0.3 (−1.9/1.3)	**−35.8 (-67.4/-4.3)**	−5.4 (−19.0/8.3)	−2.1 (−4.8/0.6	−0.4 (−1.3/0.4)	−0.21 (−0.5/0.1)	−0.6 (-1.9/0.8)	−0.6 (−1.9/0.8)
Maximum speed (km/h)	−56.9 (−118.5/4.7)	−0.3 (−0.7/0.2)	−0.7 (−8.7/7.3)	.5 (−2.9/4.0)	−0.1 (−0.7/0.7)	−0.1 (−.2/0.2)	0.01 (−0.1/0.1)	−0.02 (−.4 .3)	−0.1 (−0.5/0.2)
Accelerations (N)	−52.1 (−112.5/9.8)	**−0.5(-0.9/0-.1)**	−5.0 (−12.7/3.1)	−.5 (−4.0/2.9)	−0.01 (−.7 .7)	−0.1 (−.3/0.2)	−0.07 (−0.2/0.0)	0.1 (−0.2/0.4)	−0.3 (−0.6/0.1)
Decelerations (N)	35.9 (−17.8/90.1)	0.04 (−0.3/0.4)	−1.9 (−9.0/5.1)	−.6 (−3.7/2.4)	−0.1 (−0.8/0.5)	0.00 (−0.2/0.2)	0.00 (−0.1/0.1)	−0.1 (−0.4/0.2)	0.3 (0.0/0.6)
Jumps >40 cm (N)	12.4 (−127.9/149.0)	0.19 (−0.7/1.1)	0.6 (−17.5/18.6)	−4.2 (−2.0/3.5)	−0.9 (−2.5/0.7)	−0.3 (−0.8/0.2)	−0.02 (−0.2/0.2)	**1.0** (0.2/1.8)	0.2 (−0.6/1.0)
COD to left (N)	−15.4 (−70.8/40.4)	0.2 (−0.2/0.6)	1.5 (−5.8/8.9)	−.1 (−.3/3.0)	0.2 (−0.4/0.9)	−0.05 (−0.2/0.2)	0.02 (−0.1/0.1)	−0.02 (−0.3/0.3)	−0.2 (−0.5/0.2)
COD to right (N)	14.2 (−37.9/67.0)	−0.03 (−0.2/0.6)	−4.1 (−11.0/2.8)	**−3.2 (-6.1/-0.2)**	**−0.7 (-1.3/-0.1)**	**−0.2 (-0.4/0.0)**	**−0.09 (-0.2/0.0)**	0.1 (−0.2/0.4)	−0.03 (−0.3/0.3)
RHIE block efforts (N)	17.5 (−78.4/111.9)	0.1 (−0.6/0.7)	1.6 (−10.7/14.0)	3.4 (-2.0/8.7)	0.1 (−1.0/1.2)	0.2 (−.1/0.6)	0.06 (−0.1/0.2)	0.2 (−0.3/0.8)	0.3 (−0.2/0.9)
Average RHIE effort (N)	−9.8 (−57.4/37.6)	−0.2 (−0.5/0.2)	3.4 (−2.8/9.5)	2.4 (−0.2/5.1)	0.4 (−.2 .9)	**0.2 (0.0/0.3)**	0.06 (0.0/0.1)	−0.1 (−0.3/0.2)	0.01 (−0.2/0.3)
RHIE block recovery time (RHIE/min)	18.1 (−46.9/83.1)	−0.1 (−0.5/0.3)	**−9.1–17.4/0-.7)**	−**4.2 (-7.9/-0.6)**	−0.2 (−1.0/0.5)	**−0.3 (-0.5/-0.1)**	−0.05 (−0.1//0.0)	−0.2 (−0.5/0.2)	0.02 (−0.3/0.4)

Jumps >40 cm (N)	COD to left (N)	COD to right (N)	Explosive effort	RHIE blocks effort (N)	Average RHIE effort (N)	RHIE block recovery time (RHIE/min)	Distance >20w (m)	Distance >55w (m)	Player load
**4.4** (0.8/8.0)	**21.6 (10.7/32.4)**	**22.3** (12.2/32.3)	**83.6 (60.5/106.7)**	30.1 **(21.86/38.31)**	**4.5 (3.95 5.06)**	**2.8 (1.50 4.42)**	**2,666.0 (2,089.1/3,242.9)**	**312.4 (223.0/401.8)**	**1,166.2 (956.5/1,375.9)**
0.1 (−0.4/0.6)	0.5 (−1.1/2.0)	−1.2 (−2.7/0.3)	−1.5 (−4.8/1.7)	−0.9 (−2.08/0.35)	−0.05 (−0.13/0.03)	0.1 (−0.10/0.32)	**−204.4 (-289.6/-119.1)**	**−17.7 (-31.1/-4.4)**	−11.9 (−41.5/17.6)
**−0.9** (−1.4/-0.4)	**−1.7 (-3.1/-0.4)**	−0.5 (−1.5/0.4)	**−4.2 (-7.3/-1.1)**	−0.8 (−1.7/0.04)	−0.05 (−0.10/0.00)	0.15 (−0.03/0.32)	**84.2 (24.1/144.2)**	**−9.3 (-18.4/-0.2)**	−3.6 (−33.1/25.8)
0.1 (0.0/0.3)	−0.1 (−0.6/0.4)	0.1 (−0.6/0.4)	0.2 (−.9/1.2)	−0.3 (−0.7/0.1)	−0.02 (−0.05/0.01)	0.03 (−0.03/0.10)	−20.2 (−47.0/6.6)	**−4.2 (-8.4/-0.1)**	−2.1 (−11.8/7.6)
−0.04 (−0.4/0.3)	−0.4 (−1.4/0.7)	−0.5 (−1.5/0.5)	−1.8 (−4.0/0.4)	**−0.8 (-1.5/.01)**	0.02 (−0.04/0.07)	0.10 (−0.04/0.24)	−40.0 (−95.8/15.9)	−8.5 (−17.2/0.2)	−15.0 (−34.7/4.8)
−0.1 (−0.2/0.0)	0.2 (−0.2/0.5)	0.1 (−0.3/0.4)	0.5 (−0.3/1.2)	**0.3 (.07 .63)**	0.01 (−0.01 .03)	−0.01 (−0.06/0.04)	**37.7 (17.8/57.6)**	**6.1(3.0/9.2)**	**11.8 (4.8/18.7)**
0.00 (0.00/0.00)	0.00 (−0.0/0.02)	**−0.00** (-**0.01/0.00)**	−0.01 (−0.01/0.00)	0.00 (0.00/0.00)	0.00 (0.00/0.00)	0.00 (0.00/0.00)	−0.1 (−0.3/0.1)	0.00 (0.0/0.0)	0.0 (−0.1/0.0)
0.00 (−0.02/0.03)	−0.1 (−0.03/0.1)	0.02 (−0.1/0.1)	−0.07 (−0.2/0.1)	0.00 (−0.06/0.06)	0.00 (0.00/0.01)	0.00 (−0.02/0.01)	−3.4 (−7.9/1.0)	0.01 (−.7/0.7)	−1.3 (−2.8/0.2)
0.00 (0.00/0.01)	0.01 (−0.00/0 .02)	**0.01 (0.00/0.02)**	**0.04 (0.01/0.1)**	**0.01 (0.00/0.02)**	0.00 (0.00/0.00)	0.00 (0.00/0.00)	**1.5 (0.8/2.2)**	0.1 (0.0/0.2)	**0.3 (0.1/0.5)**
−0.01 (−0.03/0.01)	0.02 (−0.03/0.01)	−0.04 (−0.1/0.0)	**−0.1 (-0.2/-0.00)**	0.00 (−0.04/0.04)	0.00 (0.00/0.01)	0.00 (−0.01/0.00)	−2.6 (−5.6/0.3)	**0.6 (0.1/1.1)**	**−1.2 (-2.3/-0.2)**
0.00 (−0.01/0.01)	−0.01 (−0.03/0.01)	−0.01 (−0.04/0.02)	−0.02 (−0.1/0.03)	0.0 (−0.03/0.02)	0.00 (0.00/0.00)	0.00 (−0.01/0.00)	**−2.2** (−3.7/-0.7)	−0.1 (−0.3/0.1)	−0.2 (−0.7/0.3)
−0.02 (−0.1/0.0)	**0.2 (0.1/0.3)**	**0.1 (0.0/0.2)**	0.2 (−0.04/0.4)	**0.1 (0.06/0.24)**	0.00 (0.00/0.01)	−0.01 (0.00/0.00)	**10.8 (4.5/17.0)**	0.9 (0.0/1.9)	**2.8 (0.7/5.0)**
−0.01 (−0.1/0.1)	−0.1 (−0.4/0.1)	−0.1 (−0.3/0.2)	−0.3 (−0.8/0.1)	−0.1 (−0.28/0.07)	0.00 (−0.02/0.01)	0.03 (0.00/0.06)	0.9 (−11.5/13.5)	0.04 (−1.9/2.0)	2.6 (-1.8/7.0)
0.2 (−0.3/0.7)	**−3.0 (-4.5–1.5)**	−1.0 (−2.5/0.4)	−1.4 (−4.6/1.7)	**−1.6 (-2.77/-0.43)**	−0.07 (−.15/0.01)	0.17 (−0.03/0.38)	−54.2 (−136.1/27.7)	−9.8 (−22.6/3.0)	−18.6 (−47.1/9.8)
0.01 (−0.1/0.1)	0.2 (−0.2/0.5)	0.1 (−0.3/0.5)	0.1 (−0.7/0.9)	−0.1 (−0.40/0.19)	0.00 (−0.01/0.03)	0.02 (−0.04/0.06)	−18.2 (−38.9/2.6)	−1.9 (−5.1/1.3)	−3.4 (−10.6/3.9)
−0.02 (−0.1/0.1)	0.13 (−0.2/0.5)	−0.2 (−0.5/0.2)	−0.1 (−0.9/0.6)	−0.2 (−.51 .07)	−0.01 (−0.03/0.01)	0.04 (−0.02/0.09)	−19.4 (−40.0/1.2)	−1.4 (−4.6/1.8)	−4.2 (−11.4/3.0)
0.04 (−0.1/0.2)	0.1 (−0.2/0.5)	0.3 (0.04/0.6)	**0.7 (0.00/1.4)**	0.2 (−0.01/0.51)	−0.01 (−0.03/0.01)	0.00 (−0.04/0.05)	7.4 (−10.8/25.6)	0.4 (−2.4/3.2)	3.5 (−2.9/10.0)
**0.3** (0.0/0.6)	**1.6 (0.7/2.4)**	**1.2 (0.4/2.0)**	1.5 (−0.3/3.4)	0.6 (−0.04/1.30)	0.03 (−0.01/0.08)	−0.11 (−0.23/0.01)	−15.0 (−61.7/31.8)	1.0 (−6.3/8.2)	16.4 (−0.1/33.0)
−0.00 (−0.1/0.1)	0.1 (−0.3/0.4)	0.02 (−0.3/0.4)	0.05 (−0.7/0.8)	0.1 (−0.18/0.36)	−0.02 (−0.03/0.00)	−0.04 (−0.09/0.01)	1.5 (−17.4/20.4)	0.01 (−2.9/2.9)	0.9 (−5.9/7.7)
−0.02 (−0.1/0.1)	−0.1 (−0.5/0.2)	**0.3 (0.01/0.6)**	−0.3 (−1.0/0.4)	−0.2 (−0.49/0.02)	0.00 (−0.02/0.02)	**0.04 (0.00/0.09)**	−6.8 (−24.6 11.1)	**−3.6 (-6.4/-0.8)**	3.3 (−3.2/9.7)
0.08 (−0.1/0.3)	−0.3 (−0.9/0.3)	0.1 (−0.5/0.7)	0.4 (−0.9/1.7)	0.1 (-0.31/0.61)	0.01 (−0.02/0.04)	−0.01 (−0.09/0.07)	7.6 (−24.5/39.7)	.8 (−4.2/5.8)	1.7 (−9.6/13.1)
0.01 (−0.1/0.1)	0.1 (−0.2/0.3)	−0.2 (−0.5/0.1)	−0.1 (-0.7/0.5)	0.1 (−0.13/0.33)	0.01 (−0.01/0.03)	−0.03 (−0.06/0.02)	−0.6 (−16.5/15.4)	**2.8 (0.3/5.3)**	−1.8 (−7.3/3.8)
0.1 (−0.1/0.2)	−0.4 (−0.8/0.0)	−0.03 (−0.4/0.4)	0.00 (−0.8/0.8)	**−0.5 (-0.79/-0.17)**	−0.2 (−0.04/0.00)	**0.09 (0.04/0.15)**	−3.6 (−25.4/18.2)	**−4.7 (-8.1/-1.3)**	−4.3 (−11.8/3.2)

COD, changes of direction; RHIE, repeated high-intensity exercise. Bold values indicate *p* < 0.05.

## 3 Results

Player match frequency was 4 ± 2 days (ranging from 3 to 20 days), training between matches was 2.7 ± 2.7 (ranging from 1 to 31 sessions), weekly sessions were 2.2 ± 1 (ranging from 1 to 5 sessions), and home/away match performances were 260 and 238, respectively.

Mixed linear model regression (Table 2) shows that training load and contextual variables significantly influence game performance. For example, match running relative distance (m/min) was positively influenced by training variables such as amount of training between matches and distance covered >20 W kg^−1^ during training sessions. On the other hand, it is negatively influenced by training variables such as distance covered >55 W kg^−1^, large block recovery time, acceleration, and days between games, and away match. Table 2 describes those contextual variables such as match avenue, training sessions between matches, days between games, and player position has significant influence on several match performance.

The decision tree regression (DTR) model was used to identify the most important training load variables associated with match performance. In addition, the DTR model shows the extent to which match performance variables are negatively or positively affected by their most important training load variable. Because several match performance variables are affected by the location of the game and the player’s position, the DTR model was controlled for these variables when necessary.

**FIGURE 2:**
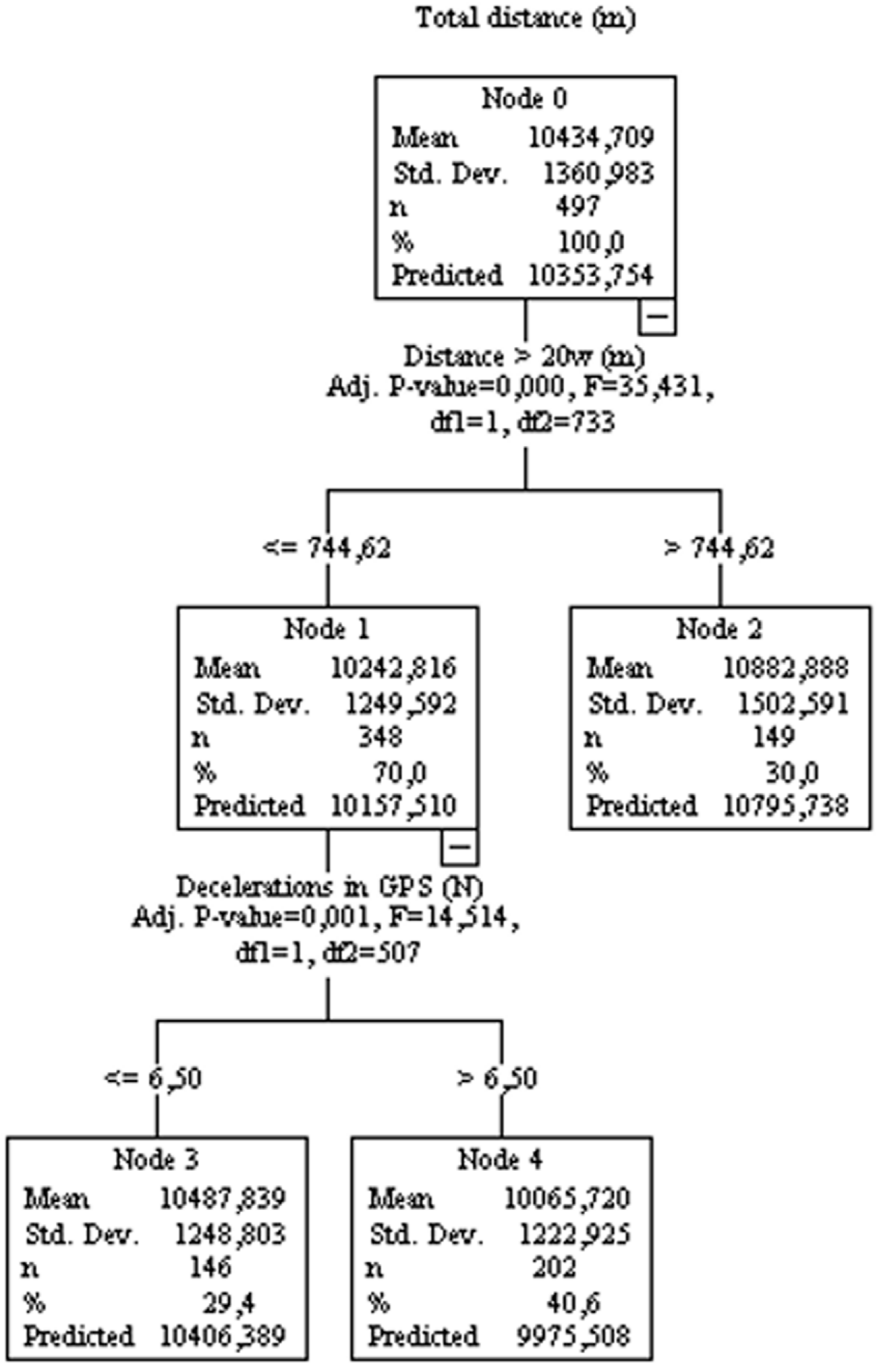
Decision tree regression for mean values of total distance controlled for Home/away condition from 77 matches from an elite team. Independent variables are data from sessions training between matches during a season.

Figure 2 (controlled for game location) shows that the total distance (shown at node zero) is associated with the amount of distance spent in activities >20 W kg^−1^ (Nodes 1 and 2). The total distance is negatively impacted by decelerations (Nodes 3 and 4). Thus, Node 1 shows that the distance covered of ≤744.6 m (at intensity >20 W kg^−1^) is associated with the predicted distance of 10,157.5 m. While running distance of >744.62 m at >20 W kg^−1^ (Node 2), the predicted distance will be statistically greater (10,795.7 m) compared to Node 1. Node 2 (with a statistically smaller total distance compared to Node 1) is divided into two more nodes (Nodes 3 and 4), indicating that in this sample subset there is a difference in performance when considering the number of decelerations performed in the training session. Thus, Node 3 indicates that performing ≤6.5 decelerations in training sessions is associated with 10,406.3 m, while Node 4 indicates that performing >6.5 decelerations in the training session is associated with 9975.5 m (statistically lower performance compared to Node 3). Here, the decision tree suggested that high running load training sessions (i.e., >744.62 m over 20 W kg^−1^) were beneficial for overall match running performance, while deceleration (i.e., >6.5) had a negative effect.

**FIGURE 3:**
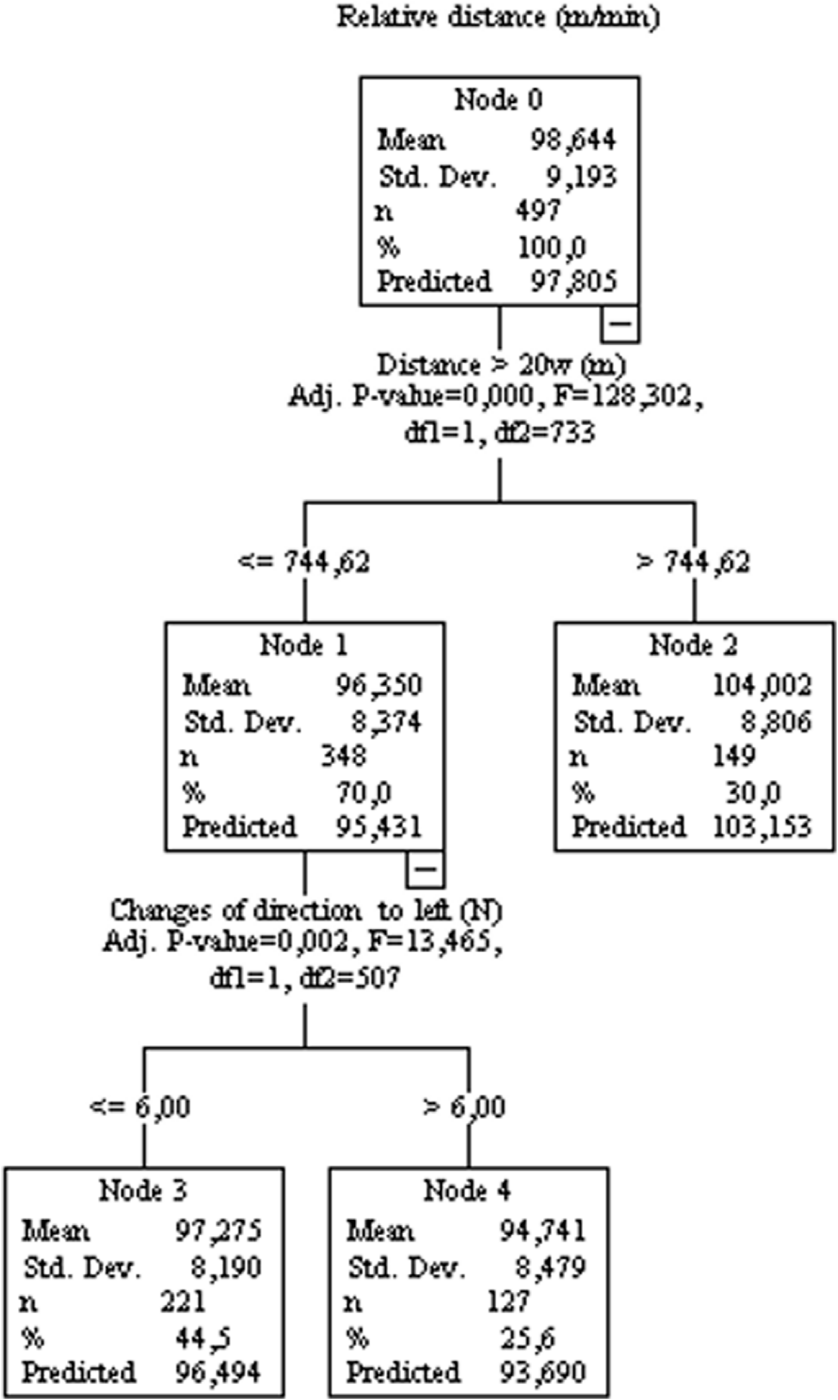
Decision tree regression for mean values of relative distance from 77 matches of an elite team. Data from training sessions between matches during a season are used as independent variables.

Figure 3 analyzes which variables influence the relative distance (controlled for game location). Hence, Node 1 shows that players who run ≤744.62 m above 20 W kg^−1^ in training sessions have a predicted relative running distance of 95.431 m/min in matches, which is statistically different from Node 2 (players who run >744.62 m above 20 W kg^−1^ between matches during the training seasons).

**FIGURE 4:**
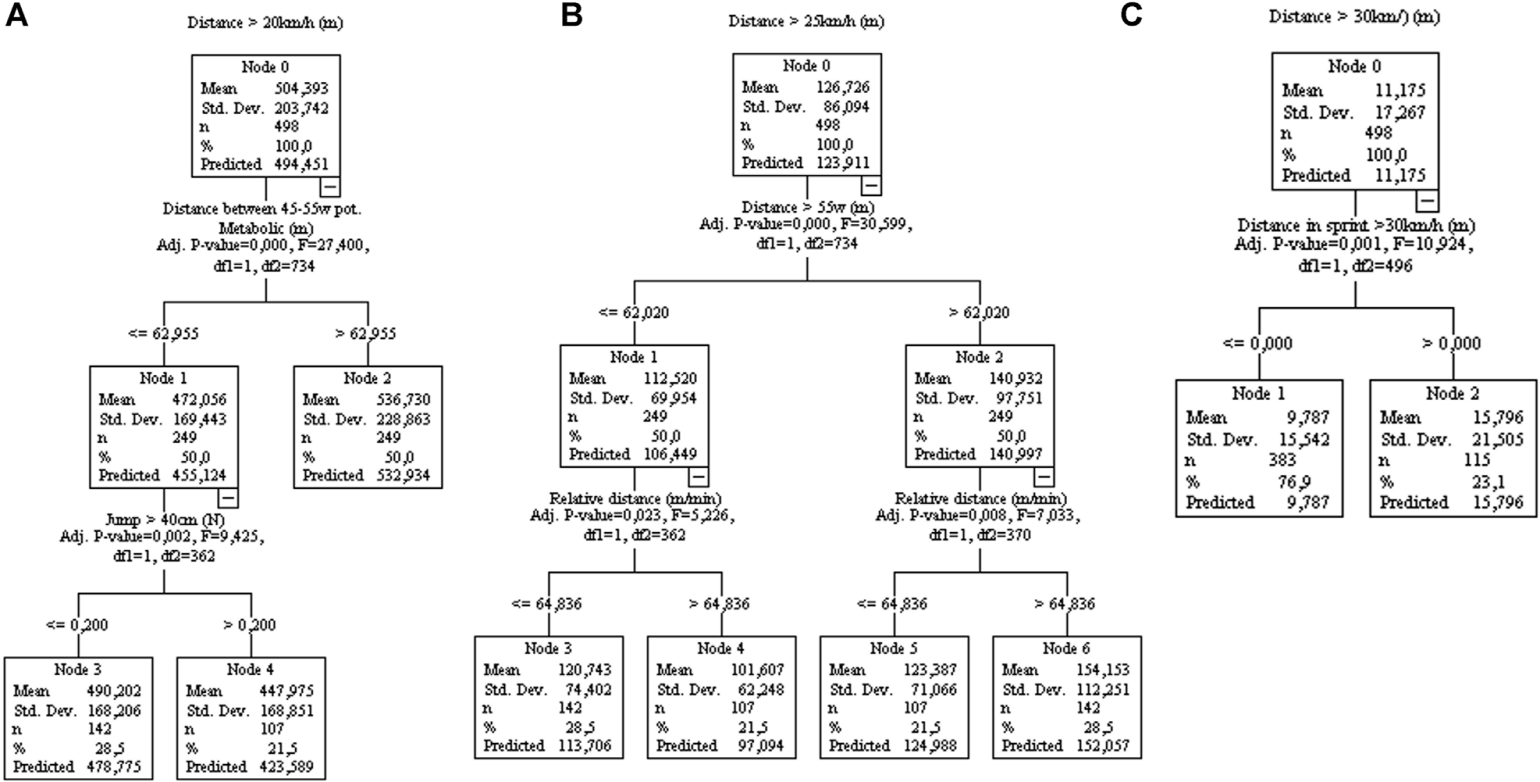
Decision tree regression for distance mean values of distance running at 20 km/h [**(A)**, controlled for home/away condition], 25 km/h [**(B)**, controlled for home/away condition], and 30 km/h **(C)** from 77 matches of an elite team. Data from training sessions between matches during a season are used as independent variables.

In Figure 4A, the total distance achieved >20 km/h in the matches is associated with 45-5Wkg^−1^ running distance activities. While running distance achieved >25 km/h and 30 km/h during matches are related to distance covered >55 W kg^−1^, and sprints during training sessions (Figures 4B, C).

**FIGURE 5:**
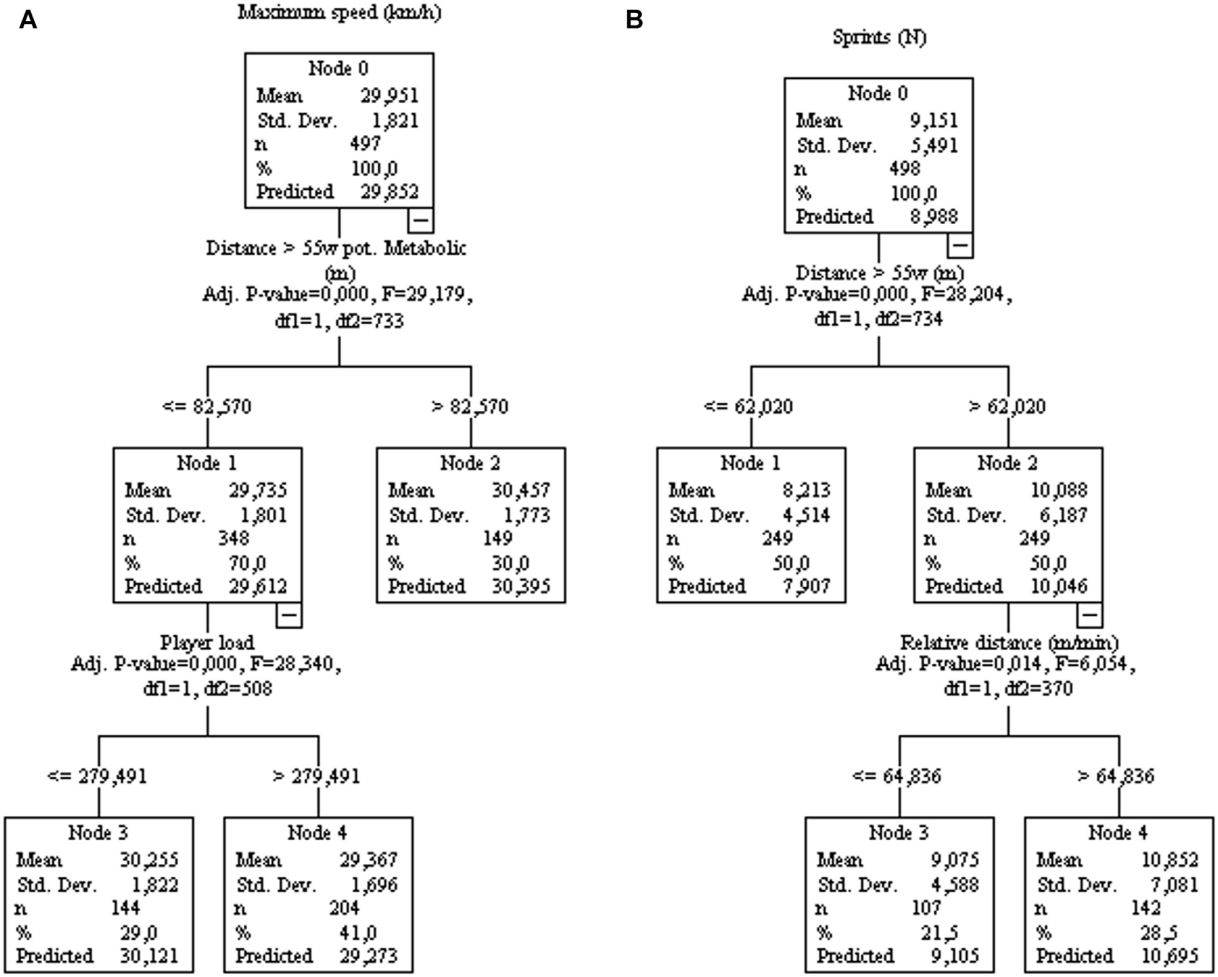
Decision tree regression for maximum speed [**(A)**, controlled for home/away condition] and amounts of sprints [**(B)**, controlled for home/away condition] from 77 matches of an elite team. Data from training sessions between matches during a season are used as independent variables.

**FIGURE 6:**
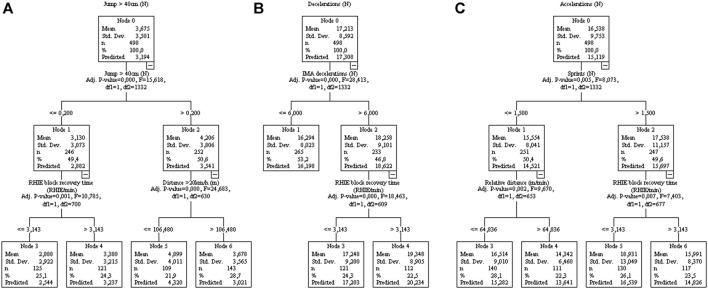
Decision tree regression for amounts of Jumps [**(A)**, controlled for home/away condition], acceleration [**(B)**, controlled for player position], and deceleration [**(C)**, controlled for player position] from 77 matches of an elite team. Data from training sessions between matches during a season are used as independent variables.

Also, the maximum speed and the number of sprints (Figures 5A, B) can be explained by the distance >55 W kg^−1^, but with interference from player load and relative distance, respectively. Likewise, the number of jumps >40 cm (Figure 6A) and decelerations (Figure 6B) were associated with it respectively variable in training sessions, but interference variables were present. Accelerations were significantly associated with sprints (Figure 6C).

**FIGURE 7:**
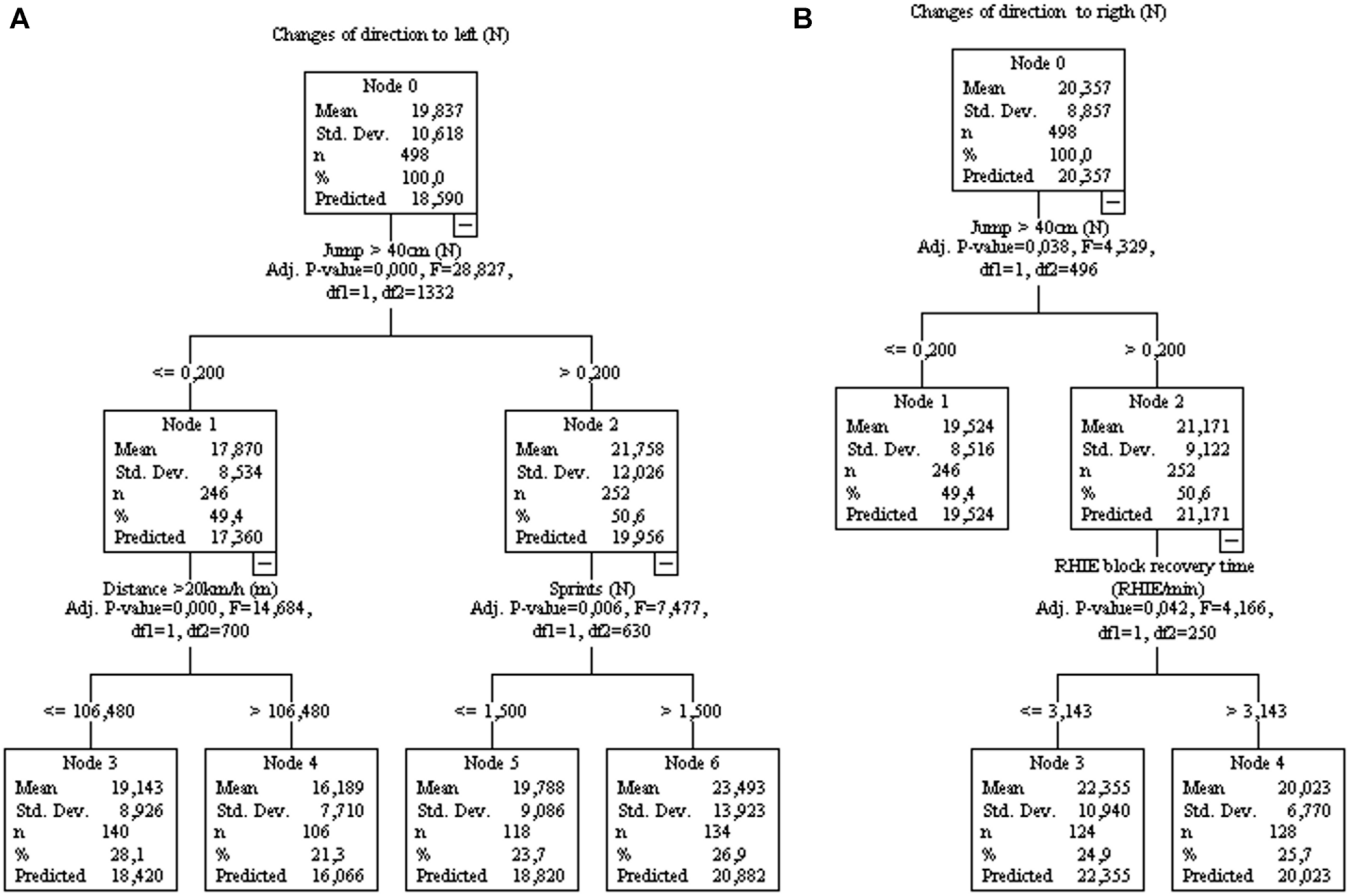
Decision tree regression for amounts of change of direction to left **(A)**, controlled for player position and to the right **(B)** from 77 matches of an elite team. Data from training sessions between matches during a season are used as independent variables.

Figures 7A, B show the greatest number of COD in the matches performed by the athletes with the highest incidence of jumps >40 cm during the training sessions. However, interference variables associated with performance improvement or decrement are present. In Figure 7A, distance >20 km/h impact negatively the COD, while sprints and RHIE block recovery time improving it.

**FIGURE 8:**
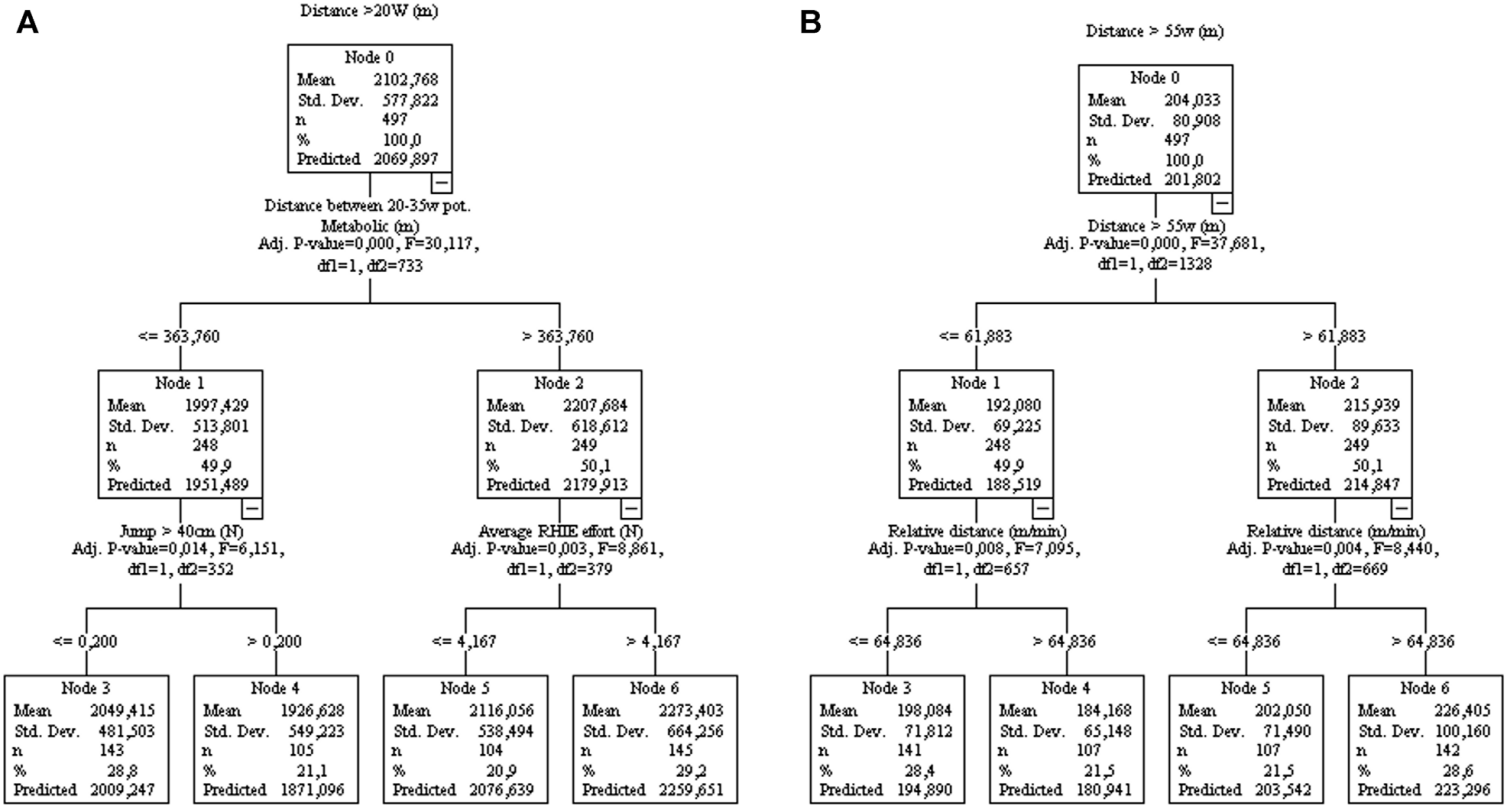
Decision tree regression for the running distance >20W [**(A)**; controlled for home/away condition] and >55W [**(B)**, controlled for player position] from 77 matches of an elite team. Data from training sessions between matches during a season are used as independent variables.

The distance running >20 or 55 W kg^−1^ was associated with the respective activity during training sessions (Figure 8).

**FIGURE 9:**
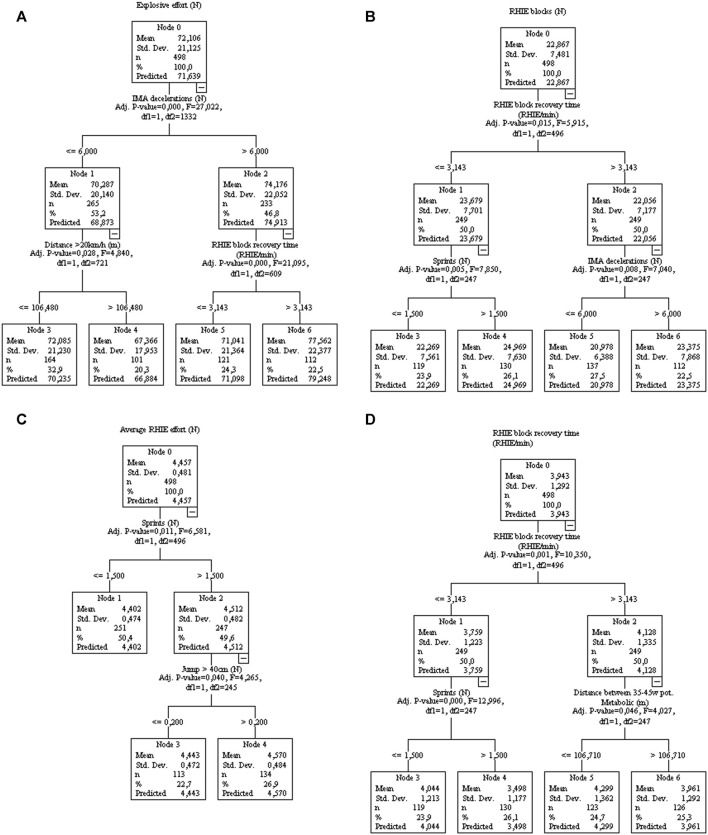
Decision tree regression for amount of explosive of effort [**(A)**, controlled for player position], repeated high-intensity effort (RHIE) block **(B)**, average RHIE **(C)**, and RHIE recovery time **(D)** from 77 matches of an elite team. Data from training sessions between matches during a season are used as independent variables.

The number of explosive efforts in matches (Figure 9A) was associated with deceleration (Nodes 1 and 2). Running distance >20 Km/h impacts the explosive effort (Nodes 3 and 4) negatively, while large RHIE block recovery time positively impacts the explosive effort (Nodes 5 and 6). RHIE block was associated with RHIE block recovery time (Figure 9B), with sprints (nodes 3 and 4) and decelerations (nodes 5 and 6) impacting positively. The average RHIE was associated with sprints and jumps (Figure 9C). RHIE block recovery time was associated with their respective training variable (Figure 9D), with the distance covered at 35–45 W kg^−1^ improving RHIE block recovery time, while sprints worsening it.

**FIGURE 10:**
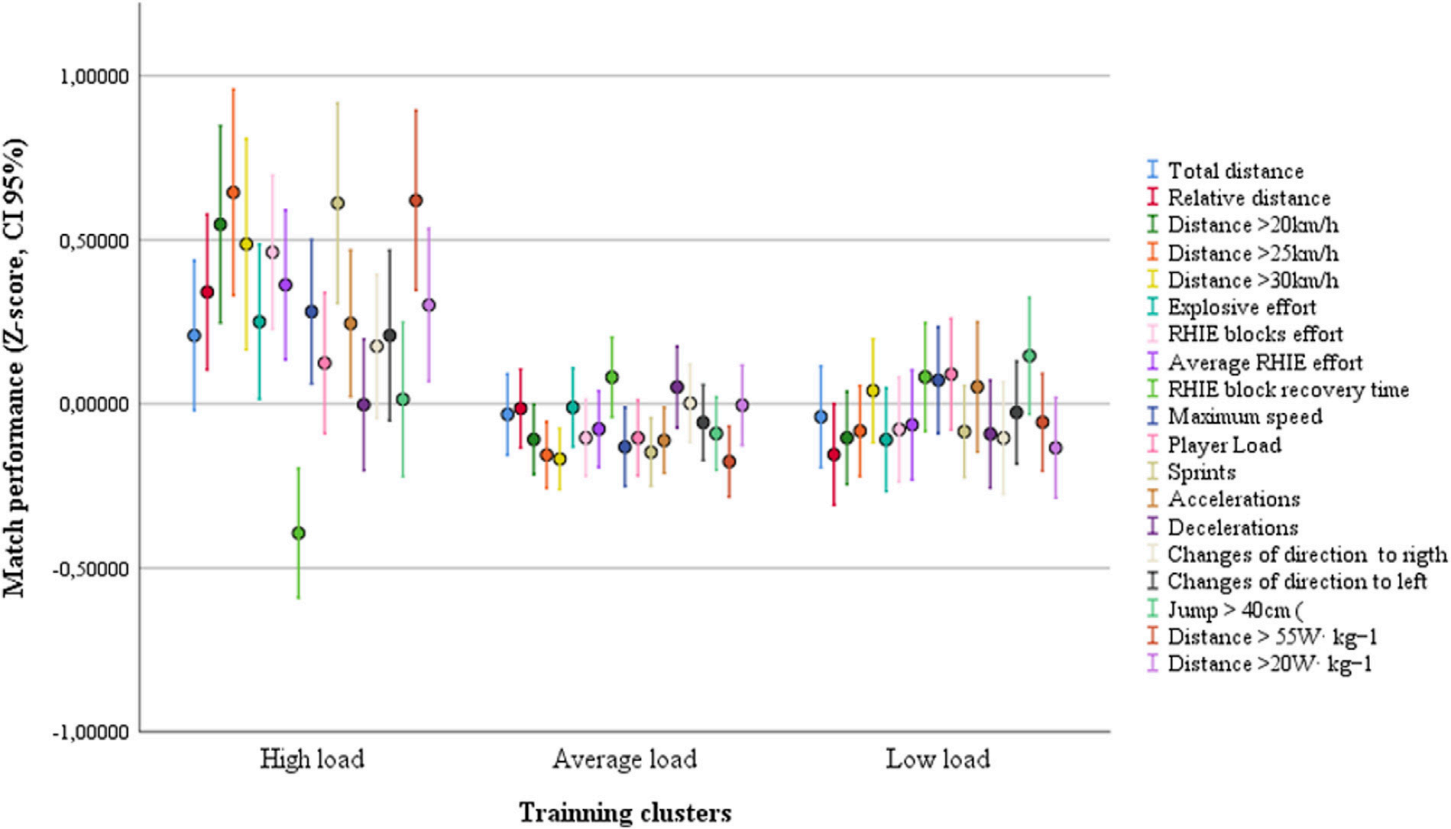
Match performance plotted as a function of three different training loads over a season from an elite Brazilian team.

**FIGURE 11:**
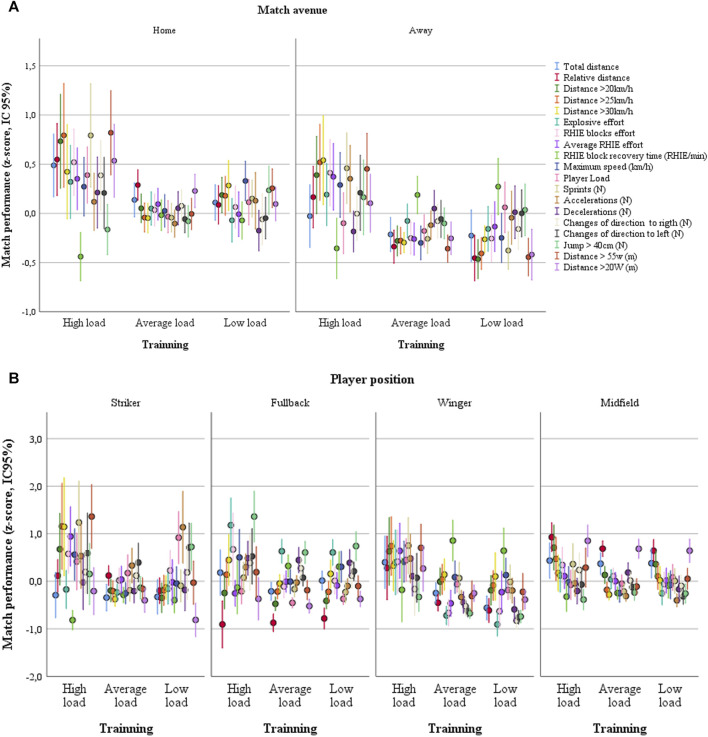
Match performance plotted as a function of training load separated by home-away match condition **(A)** and player position **(B)**.

To verify the association between training load and match performance, in Figure 10, three clusters were created from training load (high, medium, and low load clusters) and match performance (high, medium, and low performance clusters). All the variables of the three clusters from both match performances and training load are significantly different within (ANOVA: *p* < 0.001). Figure 10 plot data from match performance data as a function of training load. The Chi-square test identified a significant association (*p* < 0.001, phi = 0.246) between training load and match performance. The training load clusters were not associated with player position or home-away match condition (data not shown, all >0.325). However, the match performances clusters were associated with player position and home-away match condition (*p* = 0.001, phi = 0.364, see Figure 11).

## 4 Discussion

It is important to note that these data are from a busy season of a world elite team (77 matches, i.e., one match every 4 days per player). The team has a 36-man squad that rotates throughout the season to keep up with the match schedule. This is an extremely high volume for elite soccer, although it is common in Brazil. The main challenge for the training team is to give the athletes the physical conditioning stimulus (in a few training opportunities, i.e., two training sessions between matches) without leading them to acute overtraining (high load in one session) or chronic overtraining (high load of training combined with high load of matches), which can lead to injuries due to overload (Kalkhoven et al., 2021). An optimal training load for Brazilian elite soccer players has not been reported in the literature. Therefore, Brazilian soccer coaches/trainers certainly learn from trial and error. This work presents a case study analyzing training load values and their association with field performance. In this sense, it is indicated here the load values that will benefit athletes who are subjected to a busy calendar (such as the Brazilian). The main finding of this study was to identify that performance variables in games of a team with an extensive and congested season are related to the training load variables in a specific way. Possible stimuli interference was also identified, suggesting that a periodization (or load modulation) of the stimuli is necessary to improve the performance of athletes.

The mixed linear model and regression trees suggest that the variables from running sessions (which have a greater mechanical impact on players), such as COD, accelerations, decelerations, and jumps (McBurnie et al., 2022), negatively affect game speed variables (such as running at speeds >20, >25, or >30 km/h) and total running distance. On the other hand, variables related to greater mechanical impact positively influence the performance of variables related to their domain (COD, accelerations, decelerations, and jumps), indicating the specificity of the stimulus. The variables related to running in a straight line are positively influenced by the variables related to distances covered at high intensity (running >25 km/h or >20 W kg^−1^), which reinforces the specificity of the stimulus. For example, total distance, relative speed, or distance traveled at 20 km/h during games are all positively influenced by distances traveled >20W. In addition, covering distances >25, >30 km/h, running at maximum speed, or the number of sprints are all positively influenced by covering distances at >55 W kg^−1^ in training sessions. Previous research (Little and Williams, 2005) has shown that acceleration, agility and maximal speed are specific traits and are relatively unrelated to one another in soccer players. In this sense, the authors suggested that training procedures for each component need to be considered during the training process. For example, a previous experimental study (Young et al., 2001) showed that an increase in COD load reduces the gains in sprint performance. COD training improves the COD-test, but does not improve straight line speed performance. Therefore, the present data indicated that an interference phenomenon could occur in the real scenario, i.e., when a high load of two distinct capabilities (running in straight line vs COD, deceleration, and jump) is applied throughout the season. same training block. It is important to note that individuals with poor performance in straight-line running (always on the left side of the decision trees) are influenced by the variables COD, decelerations, jumps, or player load. For example, the total distance covered in games is positively influenced by the distance covered at >20 W kg^−1^; however, individuals who cover a distance ≤744.62 m at 20 W kg^−1^ (left side of the tree, Figure 2A) are the ones who suffer a negative influence from the high load of deceleration activities (>6.5 actions). The same pattern can be observed for running variables such as relative distance, running at different speeds (>20, >25, >30 km/h or maximal speed) or intensities >20 or 55 W kg^−1^.

In Figure 11, three match performances were plotted as a function of three training loads. Significant associations were identified between training load and performance in matches. The high load values described here have a beneficial effect on match performance, despite inter-stimuli interference discussed above. In addition, our cluster analysis revealed that there are three distinct performances in both match and training performance. As expected, the match performance cluster was significantly associated with the player position and the home-away factor (Figure 11). However, the training load was not associated with player position or the match venue. Thus, we can rule out the possibility that the association between training load and match performance found in the present study is significantly influenced by player position or the home-away factor.

In a practical application, the decision tree regression model suggests beneficial loads and possible corrections to improve athlete performance. In this sense, the training blocks to improve agility, accelerations/decelerations, and jumps must be carefully planned to improve their respective qualities on the match, and at the same time, strategies must be tested to mitigate drops in straight-line running performance. For example, match relative speed was associated with running a distance greater than 744.62 m at an intensity of >20W. However, athletes who do not reach this distance can improve their relative speed if they have a lower the activities related to mechanical impacts (e.g., COD or jumps). As these data are based on observational data, future studies experimenting with this load modulation and periodizing the stimuli may confirm our findings. Also, future studies investigating whether the interferences identified here are due to a lack of specific stimulus (due to the short training time to provide the stimuli in a busy schedule) or due to cross interference.

## 5 Conclusion

This study presents a positive training load from a busy season of an elite Brazilian professional team. In addition, it can be stated that the interference effect occurs when high physical training is applied to different physical skills (e.g., COD and running in a straight line) throughout the season. Additionally, the regression tree model can be a useful tool to identify optimal loads and possible corrections to improve the athlete’s match performance.

## Data Availability

The raw data supporting the conclusion of this article will be made available by the authors, without undue reservation.
